# Improving Data Caching of the STochastic Engine for Pathway Simulation (STEPS)

**DOI:** 10.1186/1471-2202-13-S1-P154

**Published:** 2012-07-16

**Authors:** Weiliang Chen, Erik De Schutter

**Affiliations:** 1 Computational Neuroscience Unit, Okinawa Institute of Science and Technology, Okinawa 904-0411, Japan

## 

Though the years the efficiency of Gillespie SSA [1] has been improved by different approaches. Many alternatives of the Direct method have been proposed [2-4] reducing the algorithmic complexity from O(*N*) to O(1), whilst maintaining the accuracy of the solution. Other solutions provide further speedup by introducing approximations to the system, examples include tau-leaping [5] and several forms of deterministic-stochastic hybrid methods [6]. Parallelization of SSA has also been studied and achieved some degree of success [7]. However, most of these studies do not address the actual implementation of the simulator, since it depends on not only the algorithm itself, but also less generic factors such as the type of operating platform, the programming style of developers, etc.

In CNS 2011 we've introduced STEPS 1.3.0 [8], which replaces the Direct method with Composition and Rejection solution [4]. Although the new implementation provides reasonable speedup comparing to its predecessors and outperforms several stochastic reaction-diffusion simulators that are publicly available [9], further profiling indicated that all potential benefits of the CR solution had not yet been reached, mainly due to the less efficient data structure inherited from previous versions. This raised our interest on how the detail implementation of STEPS, particularly data structure that relates to the SSA kernel, affects its overall efficiency.

We’ve identified the proportions of algorithmic cost (computational cost introduced by SSA itself) and data accessing cost (the time for caching data from memory) in a single SSA iteration simulation with STEPS 1.3.0. The result confirms our speculation that although the algorithmic cost has been reduced significantly by the new algorithm, the data accessing cost was mostly maintained, therefore becoming the major bottleneck of performance. We’ve redesigned the internal data structure according to this analysis, so that data accessed during SSA iteration is stored cohesively, and consequently more friendly for memory caching. Benchmarking test shows that the performance of STEPS is significantly improved due to better memory caching of the simulator.

In this poster I will present our profiling results, and the changes we’ve made in our new implementation. I will also present the performance comparison of both versions, showing that the detailed implementation of data structures can significantly affect the performance of a SSA-based simulator, even if the algorithm applied remains the same.
